# Recruitment, screening, and baseline participant characteristics in the WALK 2.0 study: A randomized controlled trial using web 2.0 applications to promote physical activity

**DOI:** 10.1016/j.conctc.2015.12.004

**Published:** 2015-12-21

**Authors:** Cristina M. Caperchione, Mitch J. Duncan, Richard R. Rosenkranz, Corneel Vandelanotte, Anetta K. Van Itallie, Trevor N. Savage, Cindy Hooker, Anthony J. Maeder, W. Kerry Mummery, Gregory S. Kolt

**Affiliations:** a School of Health and Exercise Sciences, University of British Columbia, Kelowna, Canada; b School of Medicine and Public Health & Priority Research Centre for Physical Activity and Nutrition, University of Newcastle, Newcastle, Australia; c Department of Human Nutrition, Kansas State University, USA; d School of Human, Health and Social Sciences, CQUniversity, Rockhampton, Australia; e School of Science and Health, Western Sydney University, Sydney, Australia; f School of Computing, Engineering and Mathematics, Western Sydney University, Sydney, Australia; g Faculty of Physical Education and Recreation, University of Alberta, Edmonton, Canada

**Keywords:** Randomized controlled trial, Recruitment, Physical activity, Screening, Internet

## Abstract

**Objective:**

To describe in detail the recruitment methods and enrollment rates, the screening methods, and the baseline characteristics of a sample of adults participating in the Walk 2.0 Study, an 18 month, 3-arm randomized controlled trial of a Web 2.0 based physical activity intervention.

**Methods:**

A two-fold recruitment plan was developed and implemented, including a direct mail-out to an extract from the Australian Electoral Commission electoral roll, and other supplementary methods including email and telephone. Physical activity screening involved two steps: a validated single-item self-report instrument and the follow-up Active Australia Questionnaire. Readiness for physical activity participation was also based on a two-step process of administering the Physical Activity Readiness Questionnaire and, where needed, further clearance from a medical practitioner.

**Results:**

Across all recruitment methods, a total of 1244 participants expressed interest in participating, of which 656 were deemed eligible. Of these, 504 were later enrolled in the Walk 2.0 trial (77% enrollment rate) and randomized to the Walk 1.0 group (n = 165), the Walk 2.0 group (n = 168), or the Logbook group (n = 171). Mean age of the total sample was 50.8 years, with 65.2% female and 79.1% born in Australia.

**Conclusion:**

The results of this recruitment process demonstrate the successful use of multiple strategies to obtain a diverse sample of adults eligible to take part in a web-based physical activity promotion intervention. The use of dual screening processes ensured safe participation in the intervention. This approach to recruitment and physical activity screening can be used as a model for further trials in this area.

## 1 Introduction

On a global scale, the majority of adults from Western nations are not meeting minimum physical activity recommendations [1]. Epidemiological evidence has clearly linked physical inactivity to a number of chronic diseases, specifically cardiovascular disease, type 2 diabetes, some cancers, obesity, poor mental health, osteoporosis/osteoarthritis, and premature mortality [2], [3], [4], [5]. Despite efforts to intervene, motivating adults to engage in more physical activity for optimal health benefit remains a public health challenge [6].

Traditionally, physical activity researchers have focused their efforts on face-to-face (individual or group-based) [7], [8], [9], telephone (voice and text messaging) [10], [11], [12], [13], [14], [15], and email-based interventions [16], [17]. Although many of these types of interventions have shown promising results for physical activity behavior change, they have a number of shortcomings. The duration of many of these interventions are of short term or have limited participant contact, resulting in minimal effectiveness and population health impact [18], [19]. For those that do report a significant physical activity effect, it is rarely maintained over a long period (one year or more post-intervention) of time [20]. In addition, many of these interventions are delivered in ways and, use strategies, that limit participant reach, access, and opportunity to participate [20]. Therefore, there is a need for innovative approaches that have the potential for long-term effectiveness and maintenance, as well as greater population reach and accessibility.

Internet-based interventions have become an increasingly popular approach for physical activity behavior change. Such approaches provide an attractive medium with the potential to support long-term interventions, reach large diverse populations, and provide unlimited access anywhere anytime to behavior change intervention components [21], [22], [23], [24]. Despite the potential benefits associated with Internet-based interventions, similar to non-Internet-based (face-to-face, telephone, email, etc.) interventions, the recruitment of participants can be extremely difficult [25], [26]. Researchers have found recruitment to be challenging in prevention programs, where participants are asked to change their lifestyle behaviors in order to prevent disease or illness. If participants do not perceive that a problem exists or will develop, they are less likely to participate in such a trial, resulting in poor recruitment [27].

Poor recruitment may result in an underpowered trial, and thus clinically relevant differences, which could impact public health practice, may be reported as statistically non-significant, Consequently, this increases the chance that the intervention will be abandoned before its true value is established [28]. For those trials which are not abandoned (which is very few), poor recruitment may lead to trials being extended in order to meet recruitment targets, resulting in increased costs, workload burden, and delay in the dissemination of an effective trial [26], [29]. In order to alleviate some of the potential problems associated with recruitment, it is important for those developing and delivering randomized control trials (RCT) to have a solid understanding of the barriers and challenges to recruitment, and strategies associated with improved recruitment. Thus, the aim of this paper is two-fold: 1) to describe, in detail, the methods of participant recruitment and screening, and 2) to describe baseline participant characteristics of those recruited into the WALK 2.0 trial, a three-arm, randomized controlled trial (RCT) to test the effectiveness of web-based physical activity interventions for increasing physical activity behavior change [30].

## 2 Methods

### 2.1 Study design

Details of the rationale, design, intervention, measures, and statistical procedures have been previously reported [30]. The study received approval from the Western Sydney University (formally University of Western Sydney) Human Research Ethics Committee (Reference Number H8767) and CQUniversity Human Research Ethics Committee (Reference Number H11/01–005) and the trial was registered with the Australian New Zealand Clinical Trials Registry (ACTRN12611000157976). The trial took place across two sites in Australia: Southwestern Sydney in New South Wales and Rockhampton in Central Queensland. Participants were randomly assigned to one of the three trial arms using equal groups random allocation performed through a computer-generated algorithm. Those assigned to the Web 1.0 group were given access to the existing 10,000 steps website which included core functionality associated with Web 1.0 applications. Those assigned to the Web 2.0 group were given access to a newly developed website which included advanced core functionality associated with Web 2.0 applications. Logbook group participants were given access to a paper-based logbook. All participants received a pedometer to self-monitor steps.

Outcome measures were assessed at baseline, 3, 12, and 18 months and included the following. Anthropometric measurements of height and weight were assessed using a Seca 700 measurement station, incorporating a stadiometer and scale in one unit and waist girth was assessed using a Seca 203/201 measurement tape and the NIH Girth protocol [31]. Physical activity was assessed by the Active Australia Questionnaire [32] and accelerometry (Actigraph GT3X© activity monitor). Overall total time (not 10 min bouts) is presented here, with accelerometer cut-points of: light activity = 100–1951 cpm, moderate activity = 1952–5724 cpm, and vigorous activity= >5725 cpm [33], [34]. Quality of life was assessed with the RAND36© item Short Form Health Survey–SF–36 [35]. Psychosocial characteristics were assessed using the core constructs of the Transtheoretical Model [36], Social Cognitive Theory [37], and the Theory of Planned Behavior [38]. Website usage and engagement was assessed via Google analytics and website database queries. Internet self-efficacy was assessed with the Internet Self-Efficacy Scale-ISES [39] Website usability and satisfaction was assessed by the System Usability Scale [40]. Demographic data, such as age, gender, geographic location, country of birth, employment, education and household income, were only collected at baseline and website usability was only assessed at the follow-up periods and thus is not reported in this paper.

### 2.2 Participant recruitment

In order to recruit a diverse range of participants across the two project sites, the project team developed a two-fold recruitment plan. The recruitment plan included use of 1) direct mail out using the Australian Electoral Commission (AEC) electoral roll, as the primary recruitment method, and 2) telephone, email and a variety of other methods to supplement the primary method.

#### 2.2.1 Primary recruitment method

The Australian Electoral Commission (AEC) is responsible for maintaining the electoral roll in Australia, where voting is compulsory for citizens over 18 years of age. The AEC electoral roll provides near-complete coverage of adult Australian citizens [41]. Access to an extract of the roll is permitted for the purposes of medical research at AEC's discretion and under strict conditions, in compliance with legislation to protect privacy. In the 2011–12 financial year only 11 requests were granted. To satisfy the strict protection policy of the AEC, a statement of compliance with the Australian privacy principles and a letter of support from the approving ethics committee was submitted to the AEC and permission to extract data was approved.

Permission was obtained from the AEC to access an extract of 14,000 names and addresses, with accompanying age and gender details, 7000 from each of the Federal electoral divisions of Capricornia (Rockhampton, QLD) and Werriwa (Southwestern Sydney, NSW). Personalized invitation letters were posted to persons on the extract, initially in three batches of 1000 to test the response rate. Subsequent batches were of 2000 letters per geographical location. If a response was not received from the individual, one follow up reminder letter was sent approximately 4–6 weeks after the first letter. To raise awareness and boost recruitment rates, 13 advertisements (7 in Sydney, 6 in Rockhampton) were placed over seven weeks in local print media to support the initial mail out invitation letter.

#### 2.2.2 Supplementary recruitment methods

Once the primary recruitment method had been exhausted, a variety of supplementary recruitment methods were undertaken simultaneously. First, an email was sent via the CQUniversity Rockhampton Campus (the local University) email list inviting approximately 700 staff to participate in the study. The first invitation was sent in November 2012 with two reminders sent approximately two weeks apart. Additionally, the Population Research Laboratory (PRL) at CQUniversity was utilized. The PRL is a research laboratory focused on health and social science research and specializing in computer-assisted telephone interviewing (CATI). This lab maintains a database of previous research participants who have indicated an interest in participating in future research. This database is drawn primarily from participants who had participated in the annually conducted Queensland Social Surveys [42] and Central Queensland Social Surveys [43]. Over two months in early 2013, 826 calls were made to PRL database participants who resided in the Rockhampton area.

Media-based outlets were also used as a supplementary recruitment method. This consisted of articles and advertisements in local community newsletters, newspapers, and a specific health services magazine distributed locally. Additionally, advertisements were placed on a local online community bulletin board (a local online newsletter with open access), as well as sent, via email, to community corporate partners and the university community at both sites.

### 2.3 Eligibility and further screening

#### 2.3.1 Eligibility

All participants were required to complete a screening questionnaire to register their interest in the project. Depending on their method of recruitment into the study, participants completed this questionnaire in either paper-based format, or using an online form. All screening questionnaires were uploaded or entered into the Project's Microsoft Access Database where a participant's eligibility was initially assessed. Participants were eligible to participate in the trial if they were 18 years of age or older, lived or worked in one of the two trial sites (Western Sydney or Rockhampton, Australia), had access to the internet, were able to read and speak English, had no previous membership of the comparative website (www.10000steps.org.au), were currently engaging in less than a half an hour (30 min) of moderate-to-vigorous (e.g., walking, running or playing sport) physical activity on five or more days of the week [44], [45], and were free from any medical contraindication that would prevent them from safely increasing their physical activity (assessed by the Physical Activity Readiness Questionnaire-PAR-Q) [46]. Participants who met the above eligibility criteria progressed to the next stage of enrollment into the study. Those who only failed the screening questionnaire due to being too active or having a medical condition progressed onto a further physical activity and medical screening process (outlined in section 2.3.2) where they had the potential to still enter the study or were excluded. Ineligible participants were informed by email, or by phone in cases where email was not possible. Fig. A illustrates participant flow through the recruitment, screening, and enrollment process.

**Fig. A:**
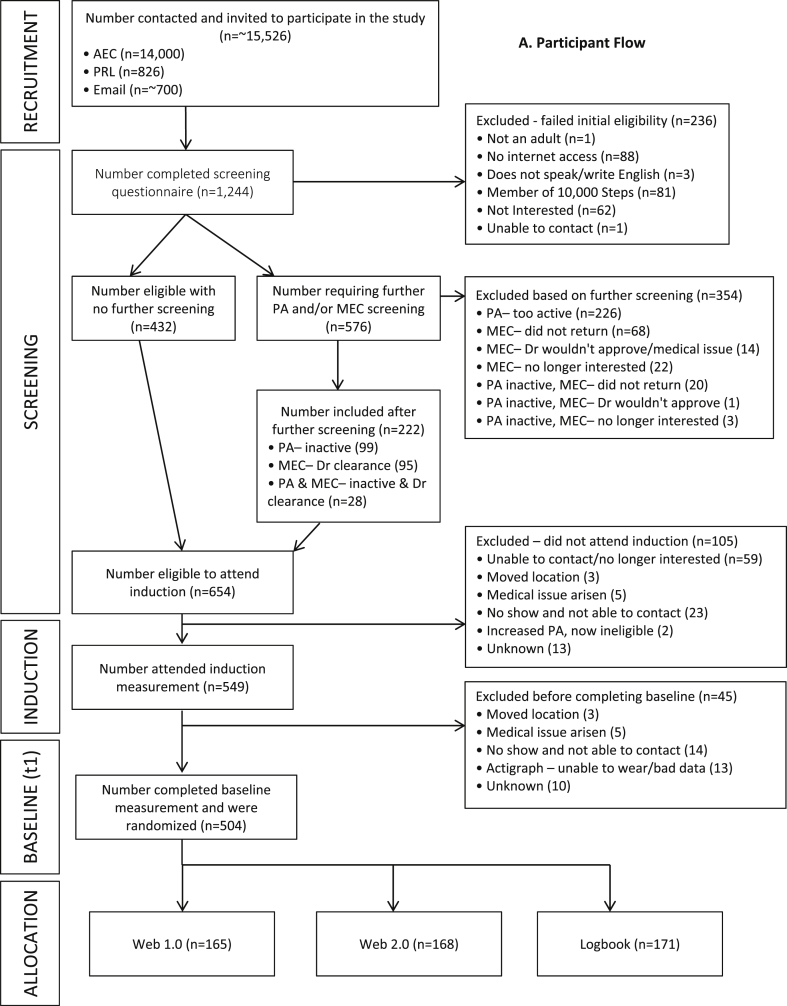
Participant flow.

#### 2.3.2 Further physical activity and medical screening

Those participants who returned a paper-based or online questionnaire and indicated they were exceeding the physical activity recommendations and/or answered yes to one of the PAR-Q questions were followed-up with a phone call by a project research assistant. During this follow-up phone call, the research assistant administered the Active Australia Questionnaire (AAQ) [32] which asked the participant about their physical activity over the last week. Those participants who responded that they completed more than 30 min of moderate or vigorous activity in 5 or more sessions were recorded as being too active and excluded from the study; the others progressed to the next stage of enrollment (Fig. A).

The research assistants also telephoned all participants who were initially ineligible due to having responded positively on the PAR-Q and provided them (via post or email) with a Medical Exercise Clearance (MEC) form. Participants were asked to take the clearance form to their General Practitioner (family physician) to gain clearance (if suitable) to participate in the study. Participants were prompted via email and phone to return these forms as soon as possible. Upon receipt of signed medical clearance form, participants were invited to commence the study and attend the induction session. Participants who failed initial eligibility based on both physical activity and medical condition were required to complete both further screening methods above.

An exception to this was those who were recruited via the PRL. Among PRL-recruited participants, those who answered YES to the single item physical activity question [45] were asked the additional AAQ questions on the phone while completing the screening questionnaire. The single item physical activity question included: ‘As a rule, do you engage in at least half an hour of moderate to vigorous exercise (such as walking or sport) on five or more days of the week? (yes/no)’ [45]. This information was then uploaded into the Access database where eligibility was assessed and they were telephoned by a research assistant if they required medical clearance (as above). Participants who were found to be eligible for the study after initial and subsequent screening were invited to attend an induction session (Fig. A). The induction session consisted of a face-to-face meeting with a research assistant at which time written consent was obtained, height, weight and waist circumference was measured, information about accelerometers was provided and accelerometers were fitted. A baseline measurement appointment (7–10 days after inductions) was also set during the induction session.

### 2.4 Data analysis

Descriptive analyses were completed and presented as means and standard deviations (SD) for continuous variables and as frequencies and proportions for categorical data. A number of categorical variables were collapsed and re-coded for reporting purposes, including education level, employment status and occupational category. Initially, education level included six categories, which were collapsed into three categories; employment status included eight categories, which were collapsed into three categories; and occupational category included nine categories, which were collapsed into five categories. Details of the original and collapsed categories for each of the three variables are outlined in Table A.

**Table A:** Demographic and anthropometric characteristics.

	Total		Web 1.0		Web 2.0		Logbook		P Value
Variable	N N	M(SD) or %	N	M(SD) or %	N	M(SD) or %	N	M(SD) or %	
**Gender**									
Male	176	34.9%	58	35.2%	54	32.1%	64	37.4%	
Female	328	65.1%	107	64.8%	114	67.9%	107	62.6%	0.592
**Age**	504	50.8 (13.1)	165	49.9 (13.5)	168	50.6 (13.4)	171	51.9 (12.3)	0.381
**Age category**									
18-34	72	14.3%	30	18.2%	22	13.1%	20	11.7%	
35-44	90	17.9%	24	14.5%	37	22.0%	29	17.0%	
45-54	137	27.2%	47	28.5%	41	24.4%	49	28.7%	0.428
55-64	128	25.4%	44	26.7%	41	24.4%	43	25.1%	
65^a^	77	15.3%	20	12.1%	27	16.1%	30	17.5%	
**Geographic location**									
Western Sydney	193	38.3%	60	36.4%	63	37.5%	70	40.9%	
Rockhampton	311	61.7%	105	63.6%	105	62.5%	101	59.1%	0.667
**Country of birth**									
Australia	398	79.0%	134	81.2%	135	80.4%	129	75.4%	
Other	106	21.0%	31	18.8%	33	19.6%	42	24.6%	0.372
**Highest education level**^a^									
Higher Education	171	33.9%	55	33.3%	57	33.9%	59	34.5%	
Trade/Diploma	193	38.3%	61	37.0%	63	37.5%	69	40.4%	0.908
School Education	140	27.8%	49	29.7%	48	28.6%	43	25.1%	
**Employment status**^b^									
Full time	234	46.4%	77	46.7%	76	45.2%	81	47.4%	
Part time/Casual	111	22.0%	38	23.0%	36	21.4%	37	21.6%	0.975
Other	159	31.5%	50	30.3%	56	33.3%	53	31.0%	
**Occupational category**^c^									
Professional	159	31.5%	50	30.3%	54	32.1%	55	32.2%	
White collar	102	20.2%	37	22.4%	36	21.4%	29	17.0%	0.656
Blue collar	31	6.2%	8	4.8%	8	4.8%	15	8.8%	
Other	53	10.5%	20	12.1%	14	8.3%	19	11.1%	
No response	159	31.5%	50	30.3%	56	33.3%	53	31.0%	
**Weekly household income**									
<$1000	140	27.8%	46	27.9%	50	29.8%	44	25.7%	
$1000 to $1999	146	29.0%	44	26.7%	43	25.6%	59	34.5%	0.574
$2000 to $5000^a^	150	29.8%	49	29.7%	52	31.0%	49	28.7%	
No response	68	13.5%	26	15.8%	23	13.7%	19	11.1%	
**Height (m)**	504	1.67 (0.1)	165	1.67 (0.1)	168	1.67 (0.1)	171	1.67 (0.1)	0.884
**Weight (kg)**	504	81.9 (18.9)	165	83.2 (18.5)	168	78.5 (17.9)	171	84.0 (19.9)	0.014*
**BMI (kg/m**^2^**)**	504	29.3 (5.9)	165	29.7 (5.9)	168	28.2 (5.5)	171	30.1 (6.1)	0.007*
**BMI category**									
Under/normal weight	122	24.2%	31	18.8%	55	32.7%	36	21.1%	
Overweight	179	35.5%	64	38.8%	60	35.7%	55	32.2%	0.007*
Obese	203	40.3%	70	42.4%	53	31.5%	80	46.8%	
**Waist category**									
Healthy	67	14.3%	19	12.3%	29	18.2%	19	12.2%	
Risky	101	21.5%	36	23.4%	33	20.8%	32	20.5%	0.480
High Risk	301	64.2%	99	64.3%	97	61.0%	105	67.3%	
**Waist girth (cm)**	469	99.9 (14.3)	154	100.8 (14.3)	159	98.1 (13.8)	156	100.9 (14.8)	0.146

*P < 0.05; M = mean, SD = standard deviation.

a Highest Education: Higher education-bachelor, graduate diploma/certificate, postgraduate; Trade/Diploma-certificate, diploma, advanced diploma; School education-high school.

b Employment: Other-unemployed, retired, student, home duties, pensioner.

c Occupation: Professional–professional, manager; White collar-community and personal service workers, clerical and administrative, sales; Blue collar-technical and trades worker, operator, driver, manual labor; Other-taxi driver, telephone interviewer, cashier, grazier, multiple jobs.

Study enrollment rate was calculated by dividing the number of participants enrolled (n = 504) by number of potentially eligible participants (n = 654). Differences in baseline characteristics between intervention groups were assessed using Chi-square tests for categorical variables and analysis of variance (ANOVA) for continuous variables, both with *p*-value of <0.05 to declare statistical significance (without adjusting for multiple comparisons). All data was managed and analyzed using SPSS version 22.

## 3 Results

### 3.1 Recruitment, screening and participant flow

The AEC mail-out (primary recruitment method) was sent to a total of 14,000 individuals across both sites, of which 714 individuals responded with interest (recruitment response rate of 5.1%) and began the screening process. A total of 283 of these individuals completed baseline measures. With regards to our supplementary recruitment methods; approximately 700 were contacted via CQU email, 66 responded to the email (recruitment response rate of 9.4%) and began the screening process and 27 completed baseline assessments; 826 were contacted via the PRL, in which 222 were screened (recruitment response rate of 26.8%) and 80 completed baseline assessment. A variety of other media-based methods (e.g., newsprint, radio) were also used, although specific data regarding how many were contacted and by which specific media they learned about WALK 2.0 are not available. We did, however, screen 242 individuals who fell into this “other media” category, of which 114 completed baseline assessments. A total of 504 participants completed baseline measures and were randomized into the WALK 2.0 trial over the 15-month enrollment period (March 29, 2012 to June 18, 2013).

Of those who completed the initial screening questionnaire (n = 1244), 236 (19.0%) were excluded for not meeting initial eligibility, 432 (34.7%) were determined to be eligible with no further screening, and invited to attend the induction appointment. The remaining 576 were required to undergo further physical activity and/or medical screening. Of the 576 who underwent further screening, a total of 354 (61.4%) were deemed ineligible and excluded from the study. The remaining 222 were determined to be eligible and were invited to attend the induction appointment. The induction appointment was attended by 83.1% (n = 549) of the 654 eligible participants (432 initially eligible + 222 determined eligible after further screening). Of the 549 who attended the induction session, 504 participants went on to complete their baseline assessment and were randomized into the Web 1.0 group (n = 165), the Web 2.0 group (n = 168), or the Logbook group (n = 171). With 654 participants potentially eligible to participate and 504 who were randomized and completed baseline assessment, our enrollment rate was 77%.

Of the 654 participants deemed eligible to participate after the comprehensive screening process 105 did not attended the induction appointment and 45 did not complete the baseline and randomization appointment. Those who were excluded (n = 105) did not attend the induction for reasons for two primary reasons, including; they were no longer interested or researchers were unable to contact (n = 59), and they booked an induction but did not show up for the appointment and were unable to be contacted (n = 23). Additional reasons are indicated in Fig. A. Of the 549 who completed the induction, a further 45 voluntarily withdrew from the trial before completing baseline primarily because they booked a baseline appointment, but did not attend the appointment and were unable to be contacted (n = 14), and participants had actigraph issues and were unable to wear them, thus presenting invalid actigraph data (n = 13). Additional reasons for voluntary withdraw are indicated in Fig. A.

### 3.2 Baseline characteristics

#### 3.2.1 Demographics and anthropometric characteristics

Among those who were randomized (n = 504), the mean age (SD) was 50.8 (13.1) years, with the majority female (65.1%), living in Rockhampton (61.7%), and born in Australia (79.0%). In terms of education level, 33.8% reported having completed higher education (e.g., Bachelor's degree, graduate or post-graduate degree) and 38.3% reported having completed vocational or technical education (e.g. College diploma, certificate). Just under half of the participants (46.4%) reported being in full-time work, with 31.5% indicating they were employed in a professional position (e.g., nurse, manager). There was a fairly even distribution across the weekly household income categories, with 27.8% of participants reporting <$1, 000 AUD week, 29% reporting $1000 to $2000 AUD per week, and 29.8% reporting $2000 to $5000 AUD per week. Chi-square analyses revealed no significant differences (*p* < 0.05) between the intervention groups for any of the demographic variables. Demographic characteristics across all three trial groups can be found in Table A.

Concerning anthropometric data, a mean (SD) height, weight and BMI of enrolled participants was 1.67 m (0.09), 81.9 kg (18.9) and 29.3 (5.9) respectively. When classifying BMI in established categories, over 75% of the total sample were classified as either overweight or obese. Mean (SD) waist girth was 99.9 cm (14.3). There were no significant between-group differences for height or waist girth, however, significant differences were reported for weight (*p* = 0.014), BMI (*p* = 0.007) and BMI categories (*p* = 0.007). Anthropometric characteristics across all three trial groups is detailed in Table A.

#### 3.2.2 Physical activity characteristics

Table B reports subjective and objective baseline physical activity outcomes as well as psychosocial characteristics including of Quality of Life, core constructs of the Transtheoretical Model, Social Cognitive Theory, and Theory of Planned Behavior, and internet self-efficacy. Based on self-reported physical activity using the AAQ, just over 50% of the sample were classified as insufficiently active and 6.3% reported no activity. In terms of self-reported minutes of moderate and vigorous physical activity, participants engaged in a mean (SD) of 36.87 (93.5) minutes and 46.63 (93.7) vigorous minutes per week. Objectively measured physical activity, assessed by the Actigraph GT3X activity monitor, showed that the average daily step count for participants was just over 7200 steps per day and that participants spent 535 min a day being sedentary. In terms of objective measured minutes of moderate-vigorous physical activity, participants engaged in a mean (SD) of 24 (20.3) minutes per day. No significant between-group differences were reported for subjectively or objectively measured physical activity.

**Table B:** Physical Activity and psycho-social characteristics.

Variable	Total		Web 1.0		Web 2.0		Logbook		P Value
	N	M(SD) or %	N	M(SD) or %	N	M(SD) or %	N	M(SD) or %	
**Self-reported physical activity**									
Weekly sessions of walking	504	5.08 (4.8)	165	4.61 (4.5)	168	5.52 (5.1)	171	5.12 (4.9)	0.224
Weekly sessions of moderate activity	504	0.84 (2.0)	165	0.66 (1.8)	168	0.91 (1.7)	171	0.95 (2.5)	0.356
Weekly sessions of vigorous activity	504	1.24 (2.2)	165	1.07 (2.0)	168	1.12 (1.6)	171	1.51 (2.8)	0.126
Weekly minutes of walking	504	126.53 (153.4)	165	115.27 (128.9)	168	129.82 (158.7)	171	134.15 (169.4)	0.500
Weekly minute of moderate activity	504	36.87 (93.5)	165	33.21 (90.5)	168	33.93 (68.5)	171	43.27 (115.2)	0.544
Weekly minutes of vigorous activity	504	46.63 (93.7)	165	42.48 (99.1)	168	39.82 (69.6)	171	57.31 (107.6)	0.189
**Physical activity classification**									
No reported activity	32	6.3%	10	6.1%	9	5.4%	13	7.6%	
Insufficient activity	256	50.8%	97	58.8%	79	47.0%	80	46.8%	0.122
Sufficient activity	216	42.9%	58	35.2%	80	47.6%	78	45.6%	
**Objective physical activity**									
Average daily number of steps	465	7247.63 (2424.3)	154	7449.06 (2529.8)	157	7162.34 (2208.2)	154	7133.16 (2528.0)	0.450
**Average daily minutes of:**									
Sedentary activity	465	535.20 (83.8)	154	535.51 (85.0)	157	537.49 (78.1)	154	532.56 (88.6)	0.873
Light physical activity	465	308.01 (72.3)	154	305.66 (71.2)	157	307.92 (71.3)	154	310.43 (74.8)	0.846
Moderate physical activity	465	23.34 (17.5)	154	25.22 (19.9)	157	22.36 (16.7)	154	22.45 (15.6)	0.263
Vigorous physical activity	465	0.64 (2.8)	154	0.54 (1.9)	157	0.80 (3.1)	154	0.57 (3.3)	0.674
**Health related quality of life**									
Physical functioning	504	85.37 (16.8)	165	84.91 (18.1)	168	85.68 (16.5)	171	85.50 (15.7)	0.908
Role physical	504	81.80 (31.6)	165	81.52 (32.2)	168	83.63 (30.7)	171	80.26 (31.9)	0.612
Role emotional	504	80.89 (32.6)	165	76.77 (35.2)	168	88.69 (24.4)	171	77.19 (35.7)	0.001*
Energy fatigue	504	55.95 (19.7)	165	55.21 (17.5)	168	57.23 (21.3)	171	55.41 (20.3)	0.587
Emotional wellbeing	504	77.37 (15.7)	165	76.68 (15.8)	168	78.98 (14.7)	171	76.44 (16.5)	0.264
Social functioning	504	86.21 (19.2)	165	85.91 (19.3)	168	88.54 (18.0)	171	84.21 (20.1)	0.112
Pain	504	78.71 (21.4)	165	76.97 (23.8)	168	79.91 (20.9)	171	79.20 (19.5)	0.427
General health	504	64.53 (20.0)	165	62.52 (18.6)	168	66.28 (21.8)	171	64.77 (19.4)	0.225
**TTM-stages of change**									
Pre-contemplation	16	3.2%	8	4.8%	3	1.8%	5	2.9%	
Contemplation	201	39.9%	66	40.0%	69	41.1%	66	38.6%	
Preparation	214	42.5%	77	46.7%	73	43.5%	64	37.4%	0.008*
Action	13	2.6%	4	2.4%	0	0.0%	9	5.3%	
Maintenance	60	11.9%	10	6.1%	23	13.7%	27	15.8%	
**Theory of planned behavior**									
Intention	504	4.02 (0.8)	165	3.97 (0.8)	168	4.04 (0.7)	171	4.05 (0.7)	0.605
Subjective norm	504	3.86 (0.8)	165	3.88 (0.8)	168	3.87 (0.8)	171	3.82 (0.8)	0.759
Perceived behavioral control	504	3.95 (0.8)	165	3.97 (0.7)	168	3.92 (0.8)	171	3.97 (0.7)	0.782
Attitudes	504	4.02 (0.6)	165	4.02 (0.6)	168	4.03 (0.6)	171	4.01 (0.6)	0.976
**Constructs of social cognitive theory**									
Outcome expectations	504	3.85 (0.5)	165	3.92 (0.5)	168	3.76 (0.6)	171	3.88 (0.5)	0.026*
Self-efficacy	504	8.63 (1.8)	165	8.55 (1.9)	168	8.71 (1.7)	177	8.64 (1.8)	0.732
Barriers, Self-efficacy	504	6.83 (1.9)	165	6.83 (2.0)	168	6.94 (1.9)	171	6.72 (2.0)	0.570
**Internet self efficacy**									
Not confident at all	14	2.8%	4	2.4%	6	3.6%	4	2.3%	
Not confident	20	4.0%	7	4.2%	3	1.8%	10	5.8%	
Neither confident or not confident	59	11.7%	18	10.9%	23	13.7%	18	10.5%	0.404
Confident	198	39.3%	74	44.8%	60	35.7%	64	37.4%	
Very confident	213	42.3%	62	37.6%	76	45.2%	75	43.9%	

*P < 0.05, M = mean, SD = standard deviation, TTM = Trans-theoretical Model, Accelerometer cut-points: light activity = 100–1951 cpm, moderate activity = 1952–5724 cpm, and vigorous activity = >5725 cpm.

#### 3.2.3 Quality of Life characteristics

Of the eight SF-36 Quality of Life components, all participants consistently reported high scores for physical functioning 85.4 (16.6) and social functioning 86.2 (19.2) and low scores for energy fatigue 56 (19.7) and general health 64.5 [20]. A significant between-group difference was reported for role emotional wellbeing (*p* = 0.001), but no other components differed between groups.

#### 3.2.4 Psycho-social characteristics

In terms of the Transtheoretical Model, the majority of participants reported being in the contemplation or preparation stage. Those in the Web 1.0 group reported the highest proportion (86.7%) at the contemplation or preparation stage, followed by the Web 2.0 (84.6%) and the Logbook group (76%). A significant between-group difference (*p* = 0.008) was reported for stages of the Transtheoretical Model. Theory of Planned behavior scores were relatively similar for all constructs, with no between-group differences. Of the Social Cognitive Theory constructs included (outcome expectations, self-efficacy, and barriers self-efficacy) a significant between-group difference was reported for outcome expectations (*p* = 0.026), but no differences were shown for self-efficacy or barriers self-efficacy.

#### 3.2.5 Internet self-efficacy characteristics

Internet self-efficacy outcomes indicated that the majority of the total sample (81.6%) was either confident or very confident in using the internet. No significant between-group differences were found for Internet self-efficacy.

## 4 Discussion

This paper details the WALK 2.0 recruitment and screening methods, reports on the study enrollment rate, and describes participant baseline characteristics. The recruitment strategies employed for WALK 2.0 were diverse and included a wide reach, providing an opportunity to recruit those who may not be especially interested in participating in such a project. The use of the AEC as a primary recruitment strategy provided an avenue to recruit from a variety of areas throughout both sites, regardless of socio-economic class, ethnicity, gender, education status or income level. Furthermore, the addition of our supplementary recruitment strategies allowed us to further extend our reach as well as build community awareness about WALK 2.0 given the extensive use of local media outlets. With reference to our recruitment response rates, the use of the PRL showed to be the most effective recruitment (recruitment response rate 26.8%) strategy, however, this approach alone should be used with caution as many of these previous research participants may be considered highly motivated and might already be physically active. Research supports the use of pursuing a variety of strategies [47], as we have in the current study, as this approach provides a greater range for recruiting a community-based sample, which is a vital component for improving generalizability and external validity of a health promotion intervention [48], [49], [50].

In the past, researchers have acknowledged the importance of recruiting a representative sample for real-world generalizability, however, circumstances surrounding pressures of meeting recruitment targets and limitations surrounding ethical approval often take precedence and thus convenient recruitment approaches are undertaken, often resulting in an unrepresentative sample [50], [51], [52]. The variety of recruitment methods used for WALK 2.0 were strategically chosen for their potential to recruit a diverse, representative sample, however, we are aware that given the limited geographic locations used in WALK 2.0 (Southwestern Sydney and Rockhampton) our sample may not be representative of the Australian adult population. Thus, when replicating this study, researchers should consider utilizing methods such as the AEC or similar across a number of geographic locations (e.g. urban, regional and rural areas throughout a number of states/provinces/regions) in an attempt to recruit a representative sample. Although the modest enrollment rate associated with this method should be noted and appropriately planned for.

Furthermore, researchers have indicated that samples need to be more diverse, targeting those who represent ethnic minorities, underserved, low income, and any other priority groups who experience access, opportunity and economic barriers to physical activity participation [53]. The goal of our recruitment was diversity, and as such we employed recruitment approaches (AEC and supplementary) that had the potential to reach various populations in an attempt to recruit a diverse sample. As a result, our baseline measures display diversity within our sample in that we were able to recruit fairly equal numbers across education levels and weekly household income categories. Moreover, 20% of participants in our sample were not born in Australia, with the majority of these participants from ethnic minorities. This percentage is quite high in comparison to other physical activity interventions not specifically targeting ethnic minorities [53], [54], [55]. Our sample, however, also consisted of more women, those within the age range of 45–64, and the majority categorized as a professional or white collar worker, a similar profile often reported for physical activity and health promotion research [56].

Although there was some success in recruiting a diverse sample for WALK 2.0, more effort is needed in order to increase diversity in RCT samples, particularly with hard-to-reach populations such as men [25], [29], [57] or those in the lowest income categories [58], [59], [60]. Researchers have suggested that specific tailored and targeted recruitment strategies should be employed in order to reach these populations [47], [61]. For instance, targeting male-oriented settings such as male-dominated workplaces, sport venues, and other areas where men often socialize (e.g., Men's SHED programs, Rotary centers, Men-specific social media, etc.) has been found to be a successful approach to recruiting men to health promotion intervention programs [55], [60], [62], [63].

In addition to recruiting a diverse, real-world sample, we also wanted to target participants who would benefit most from such an intervention program, specifically those who were recognized as inactive. To help ensure this, we employed a comprehensive, two-step self-report screening process, inclusive of double physical activity screening and further medical screening if necessary. This screening process reduced our study sample by over 50%, mainly as a result of activity behaviors, which meant that a large proportion of those who were interested where too active to participate and thus ineligible to be enrolled in the project. Often times, large community based physical activity interventions attract individuals who do not necessarily represent the intended target population (i.e., those who are recognized as inactive) [64]. Research has suggested that a main reason for this is that many of these individuals are more motivated to participate in such programs possibly due to increased confidence and self-efficacy [65], [66] given that they are already sufficiently active – defined as engaging in mod–vigorous activity for 30 min a day on 5 or more days of the week [32]. Although our sample did include 42.9% who were recognized as sufficiently active at baseline, this percentage is well below other population and community level physical activity research where 80% of the sample has been classified as sufficiently active at baseline [64], [67]. Thus, by employing a two-step screening process, such as the one we have here, we were able to screen out many individuals recognized as sufficiently active, however, we were unsuccessful in recruiting a larger proportion of our target population, those who were physically inactive. Being able to recruit a sample that is largely inactive remains a challenge for population and community level physical activity interventions, and thus requires careful consideration of how participants are recruited and then screened prior to study enrollment.

## 5 Conclusions

Employing a recruitment approach that has the potential to reach diverse populations, (inclusive of those across a number of income and occupational categories, education levels, adult age categories, gender and ethnic groups) is vital to establishing a diverse sample. This will entail innovative, yet focused recruitment strategies in order to extend this reach. Our use of the AEC and supplementary strategies provided us with the opportunity to do this as the AEC provided a large, diverse selection of Australian adults across the two study sites. Although this resulted in some variability within our sample, more work is required to ensure greater diversity and representation, particularly concerning hard-to-reach populations such as men, those from the lowest income levels, eldest adults, and those for ethnic minorities. In addition, comprehensive screening of participants is essential in order to ensure that the intervention is reaching those who will benefit from it the most, specifically those who are classified as physically inactive. Our recruitment and screening was partially successful in comparison to others, however, further consideration of alternative screening methods is required in order to recruit and enroll larger proportions of inactive participants.

## References

[1] Hallal P.C., Andersen L.B., Bull F.C., Guthold R., Haskell W., Ekelund U. (2012 Jul 21). Global physical activity levels: surveillance progress, pitfalls, and prospects. Lancet.

[2] Shortreed S.M., Peeters A., Forbes A.B. (2013 May). Estimating the effect of long-term physical activity on cardiovascular disease and mortality: evidence from the Framingham Heart Study. Heart.

[3] Matthews C.E., George S.M., Moore S.C., Bowles H.R., Blair A., Park Y. (2012 Feb). Amount of time spent in sedentary behaviors and cause-specific mortality in US adults. Am. J. Clin. Nutr..

[4] US Department of Health and Human Services (2008). Physical Activity Guidelines for Americans: Be Active, Healthy and Happy.

[5] Penedo F.J., Dahn J.R. (2005 Mar). Exercise and well-being: a review of mental and physical health benefits associated with physical activity. Curr. Opin. Psychiatry.

[6] Trost S.G., Blair S.N., Khan K.M. (2014 Feb). Physical inactivity remains the greatest public health problem of the 21st century: evidence, improved methods and solutions using the '7 investments that work' as a framework. Br. J. Sports Med..

[7] Richards J., Hillsdon M., Thorogood M., Foster C. (2013). Face-to-face interventions for promoting physical activity. Cochrane Database Syst. Rev..

[8] Amiri Farahani L., Asadi-Lari M., Mohammadi E., Parvizy S., Haghdoost A.A., Taghizadeh Z. (2015). Community-based physical activity interventions among women: a systematic review. BMJ Open.

[9] Han Y., Yan J. (2014 Apr). The effect of face-to-face interventions in promoting physical activity. Am. J. Nurs..

[10] Hammerback K., Felias-Christensen G., Phelan E.A. (2012). Evaluation of a telephone-based physical activity promotion program for disadvantaged older adults. Prev. Chronic Dis..

[11] Marcus B.H., Napolitano M.A., King A.C., Lewis B.A., Whiteley J.A., Albrecht A. (2007 Jul). Telephone versus print delivery of an individualized motivationally tailored physical activity intervention: project STRIDE. Health Psychol..

[12] Opdenacker J., Boen F. (2008 Nov). Effectiveness of face-to-face versus telephone support in increasing physical activity and mental health among university employees. J. Phys. Act. Health.

[13] Buchholz S.W., Wilbur J., Ingram D., Fogg L. (2013 Aug). Physical activity text messaging interventions in adults: a systematic review. Worldv. Evid. Based Nurs..

[14] Goode A.D., Reeves M.M., Eakin E.G. (2012 Jan). Telephone-delivered interventions for physical activity and dietary behavior change: an updated systematic review. Am. J. Prev. Med..

[15] Fjeldsoe B.S., Miller Y.D., O'Brien J.L., Marshall A.L. (2012). Iterative development of MobileMums: a physical activity intervention for women with young children. Int. J. Behav. Nutr. Phys. Act..

[16] Parrott M.W., Tennant L.K., Olejnik S., Poudevigne M.S. (2008 Jul). Theory of planned behavior: implications for an email-based physical activity intervention. Psychol. Sport Exerc..

[17] Hatchett A., Hallam J.S., Ford M.A. (2013 Apr). Evaluation of a social cognitive theory-based email intervention designed to influence the physical activity of survivors of breast cancer. Psychooncology.

[18] Orrow G., Kinmonth A.L., Sanderson S., Sutton S. (2012). Effectiveness of physical activity promotion based in primary care: systematic review and meta-analysis of randomised controlled trials. BMJ.

[19] Kahn E.B., Ramsey L.T., Brownson R.C., Heath G.W., Howze E.H., Powell K.E. (2002 May). The effectiveness of interventions to increase physical activity. A systematic review. Am. J. Prev. Med..

[20] Smith B.J. (2004 Apr). Promotion of physical activity in primary health care: update of the evidence on interventions. J. Sci. Med. Sport.

[21] Joseph R.P., Durant N.H., Benitez T.J., Pekmezi D.W. (2014 Jan). Internet-based physical activity interventions. Am. J. Lifestyle Med..

[22] van den Berg M.H., Schoones J.W., Vliet Vlieland T.P. (2007). Internet-based physical activity interventions: a systematic review of the literature. J. Med. Internet Res..

[23] Marcus B.H., Nigg C.R., Riebe D., Forsyth L.H. (2000 Aug). Interactive communication strategies: implications for population-based physical-activity promotion. Am. J. Prev. Med..

[24] Barak A., Klein B., Proudfoot J.G. (2009 Aug). Defining internet-supported therapeutic interventions. Ann. Behav. Med..

[25] Tate D.F., LaRose J.G., Griffin L.P., Erickson K.E., Robichaud E.F., Perdue L. (2014). Recruitment of young adults into a randomized controlled trial of weight gain prevention: message development, methods, and cost. Trials.

[26] Treweek S., Wilkie E., Craigie A.M., Caswell S., Thompson J., Steele R.J. (2013). Meeting the challenges of recruitment to multicentre, community-based, lifestyle-change trials: a case study of the BeWEL trial. Trials.

[27] Loria C.M., Signore C., Arteaga S.S. (2010 Feb). The need for targeted weight-control approaches in young women and men. Am. J. Prev. Med..

[28] Treweek S., Lockhart P., Pitkethly M., Cook J.A., Kjeldstrom M., Johansen M. (2013). Methods to improve recruitment to randomised controlled trials: Cochrane systematic review and meta-analysis. BMJ Open.

[29] Watson J.M., Torgerson D.J. (2006). Increasing recruitment to randomised trials: a review of randomised controlled trials. BMC Med. Res. Methodol..

[30] Kolt G.S., Rosenkranz R.R., Savage T.N., Maeder A.J., Vandelanotte C., Duncan M.J. (2013). WALK 2.0 study protocol: using web 2.0 applications to promote health-related physical activity – a randomised controlled trial. BMC Public Health.

[31] National Heart Lung and Blood Institute (1998). Clinical guidelines on the identification, evaluation, and treatment of overweight and obesity in adults: the evidence report. NHLBI obesity education initiative expert panel on the identification, evaluation, and treatment of obesity in adults (US). National Heart Lung and Blood Institute.

[32] Australian Institute of Health and Welfare (2003). The Active Australia Survey: a Guide and Manual for Implementation, Analysis and Reporting.

[33] Freedson P.S., Melanson E., Sirard J. (1998 May). Calibration of the computer science and applications, inc. accelerometer. Med. Sci. Sports Exerc.

[34] Healy G.N., Matthews C.E., Dunstan D.W., Winkler E.A., Owen N. (2011 Mar). Sedentary time and cardio-metabolic biomarkers in US adults: NHANES 2003-06. Eur. Heart J..

[35] Stevenson C. (1996). SF-36: Interim Norms for Australian Data.

[36] Prochaska J.O., DiClemente C.C. (1992). Stages of change in the modification of problem behaviors. Prog. Behav. Modif..

[37] Bandura A. (1986). Social Foundations of Thought and Action: a Social Cognitive Theory.

[38] Ajzen I. (Dec. 1991). The theory of planned behavior. Organ Behav. Hum..

[39] Eastin M.S., LaRose R. (2000). Internet self efficacy and the psychology of the digital divide. J. Comput. Med. Commun..

[40] Brooke J., Jordan P.W., Thomas B., Weerdmeester B.A., McClelland A.L. (1996). SUS – a quick and dirty usability scale. Usability Evaluation in Industry.

[41] Australian Electoral Commission (2012). Australian Electoral Commission Annual Report.

[42] Caperchione C.M., Duncan M.J., Mummery W.K., Steele R., Schofield G. (2008). Mediating relationship between body mass index and the direct measures of the theory of planned behaviour on physical activity intention. Psychol. Health Med..

[43] Duncan M.J., Vandelanotte C., Caperchione C., Hanley C., Mummery W.K. (2012). Temporal trends in and relationships between screen time, physical activity, overweight and obesity. BMC Public Health.

[44] Elley C.R., Kerse N., Arroll B., Robinson E. (2003). Effectiveness of counselling patients on physical activity in general practice: cluster randomised controlled trial. Brit Med. J..

[45] Rose S.B., Elley C.R., Lawton B.A., Dowell A.C. (2008 Jan 25). A single question reliably identifies physically inactive women in primary care. N. Z. Med. J..

[46] Canadian Society for Exercise Physiology (2012). PAR-q & You. http://www.csep.ca/cmfiles/publications/parq/par-q.pdf.

[47] Foster C.E., Brennan G., Matthews A., McAdam C., Fitzsimons C., Mutrie N. (2011). Recruiting participants to walking intervention studies: a systematic review. Int. J. Behav. Nutr. Phys. Act..

[48] Victora C.G., Habicht J.P., Bryce J. (2004 Mar). Evidence-based public health: moving beyond randomized trials. Am. J. Public Health.

[49] Russo R., Coultas D., Ashmore J., Peoples J., Sloan J., Jackson B.E. (2015 Mar). Chronic obstructive pulmonary disease self-management activation research trial (COPD-SMART): results of recruitment and baseline patient characteristics. Contemp. Clin. Trials.

[50] Gheorghe A., Roberts T., Hemming K., Calvert M. (2015 Jun 12). Evaluating the generalisability of trial results: introducing a centre- and trial-level generalisability index. Pharmacoeconomics.

[51] Duncan M., Vandelanotte C., Kolt G.S., Rosenkranz R.R., Caperchione C.M., George E.S. (2014). Effectiveness of a web- and mobile phone-based intervention to promote physical activity and healthy eating in middle-aged males: randomized controlled trial of the ManUp study. J. Med. Internet Res..

[52] Hubbard G., Campbell A., Davies Z., Munro J., Ireland A., Leslie S. (2015). Experiences recruiting to a pilot trial of Cardiac Rehabilitation in patients with bowel cancer (CRIB) with an embedded process evaluation: lessons learned to improve recruitment. Pilot Feasibility Stud..

[53] Jennings C.A., Vandelanotte C., Caperchione C.M., Mummery W.K. (2014 Mar). Effectiveness of a web-based physical activity intervention for adults with Type 2 diabetes-a randomised controlled trial. Prev. Med..

[54] Frierson G.M., Williams D.M., Dunsiger S., Lewis B.A., Whiteley J.A., Albrecht A.E. (2008). Recruitment of a racially and ethnically diverse sample into a physical activity efficacy trial. Clin. Trials.

[55] Duncan M.J., Vandelanotte C., Rosenkranz R.R., Caperchione C.M., Ding H., Ellison M. (2012). Effectiveness of a website and mobile phone based physical activity and nutrition intervention for middle-aged males: trial protocol and baseline findings of the ManUp Study. BMC Public Health.

[56] Hill J.O., Wyatt H.R., Peters J.C. (2012 Jul 3). Energy balance and obesity. Circulation.

[57] Caperchione C.M., Vandelanotte C., Kolt G.S., Duncan M., Ellison M., George E. (2012 Nov). What a man wants: understanding the challenges and motivations to physical activity participation and healthy eating in middle-aged Australian men. Am. J. Mens. Health.

[58] Bukman A.J., Teuscher D., Feskens E.J., van Baak M.A., Meershoek A., Renes R.J. (2014). Perceptions on healthy eating, physical activity and lifestyle advice: opportunities for adapting lifestyle interventions to individuals with low socioeconomic status. BMC Public Health.

[59] Pescud M., Pettigrew S., Wood L., Henley N. (2014). Insights and recommendations for recruitment and retention of low socio-economic parents with overweight children. Int. J. Soc. Res. Method.

[60] Caperchione C.M., Sharp P., Bottorff J.L., Stolp S., Oliffe J.L., Johnson S.T. (2015 Jul 14). The POWERPLAY workplace physical activity and nutrition intervention for men: Study protocol and baseline characteristics. Contemp. Clin. Trials.

[61] McCann J., Ridgers N.D., Carver A., Thornton L.E., Teychenne M. (2013 Aug). Effective recruitment and retention strategies in community health programs. Health Promot J. Aust..

[62] Morgan P.J., Collins C.E., Plotnikoff R.C., McElduff P., Burrows T., Warren J.M. (2010). The SHED-IT community trial study protocol: a randomised controlled trial of weight loss programs for overweight and obese men. BMC Public Health.

[63] Wyke S., Hunt K., Gray C.M., Fenwick E., Bunn C., Donnan P.T. (2015 Jan). Football Fans in Training (FFIT): a Randomised Controlled Trial of a Gender-sensitised Weight Loss and Healthy Living Programme for Men – End of Study Report.

[64] George E.S., Rosenkranz R.R., Kolt G.S. (2013). Chronic disease and sitting time in middle-aged Australian males: findings from the 45 and Up Study. Int. J. Behav. Nutr. Phys. Act..

[65] Artinian N.T., Fletcher G.F., Mozaffarian D., Kris-Etherton P., Van Horn L., Lichtenstein A.H. (2010 Jul 27). Interventions to promote physical activity and dietary lifestyle changes for cardiovascular risk factor reduction in adults: a scientific statement from the American Heart Association. Circulation.

[66] Bandura A. (1997). Self-efficacy: The Exercise of Control.

[67] Rosenkranz R.R., Duncan M.J., Rosenkranz S.K., Kolt G.S. (2013). Active lifestyles related to excellent self-rated health and quality of life: cross sectional findings from 194,545 participants in The 45 and Up Study. BMC Public Health.

